# Idiopathic Hypertrophic Pachymeningitis Presenting as Cavernous Sinus Syndrome Mimicking Orbital Apex Pathology: A Diagnostic Challenge

**DOI:** 10.7759/cureus.111834

**Published:** 2026-06-30

**Authors:** Jeeva Shaji

**Affiliations:** 1 Critical Care Department, Vijaya Viva Hospital, Kottarakara, Kollam, IND

**Keywords:** cavernous sinus, cranial neuropathy, dural thickening, headache with neurological deficits, idiopathic hypertrophic pachymeningitis, immunosuppression, ophthalmoplegia, orbital epex syndrome, steroid dependent, steroid therapy

## Abstract

Idiopathic hypertrophic pachymeningitis (IHP) is a rare chronic fibro-inflammatory disorder characterized by localized or diffuse thickening of the dura mater. Because its clinical and radiological manifestations overlap with infectious, autoimmune, neoplastic, and vascular disorders, diagnosis is often challenging. We report a 43-year-old woman who presented with progressive left frontotemporal headache followed by diplopia, ptosis, facial numbness, and ophthalmoplegia. Neurological examination localized the lesion to the left cavernous sinus region. Contrast-enhanced magnetic resonance imaging demonstrated enhancing dural thickening involving the left cavernous sinus and adjacent temporal dura. Extensive investigations excluded infectious, neoplastic, and autoimmune etiologies. A diagnosis of IHP was established after multidisciplinary evaluation. The patient demonstrated significant clinical improvement following corticosteroid therapy. This case highlights the importance of early magnetic resonance imaging, careful neuroanatomical localization, and systematic exclusion of secondary causes when evaluating painful cranial neuropathies.

## Introduction

Idiopathic hypertrophic pachymeningitis (IHP) is a rare inflammatory disorder characterized by chronic fibrosing thickening of the dura mater, leading to the compression of the adjacent neurovascular structures, resulting in cranial nerve palsies [1]. Patients may present with persistent headache, cranial neuropathies, cerebellar dysfunction, visual disturbances, or other focal neurological deficits depending on the anatomical location of dural involvement. Similar clinical and radiological findings may occur in infections, autoimmune diseases, granulomatous disorders, neoplasms, and immunoglobulin G4-related disease, so IHP remains a diagnosis of exclusion [2].

Involvement of the cavernous sinus region may result in multiple cranial neuropathies affecting cranial nerves III, IV, VI, and the ophthalmic division of cranial nerve V, producing painful ophthalmoplegia, diplopia, ptosis, and facial sensory symptoms. Because these manifestations closely resemble cavernous sinus syndrome, orbital apex pathology, Tolosa-Hunt syndrome, and neoplastic processes, diagnosis can be difficult and often delayed.

We report a patient with IHP presenting as cavernous sinus syndrome mimicking orbital apex pathology. The case highlights the importance of neuroanatomical localization, magnetic resonance imaging, and systematic exclusion of secondary causes in establishing the diagnosis.

## Case presentation

A 43-year-old woman with history of hypothyroidism and essential hypertension presented with a six-week history of progressive left-sided frontotemporal headache. The headache was persistent, non-pulsatile, and gradually increased in severity. One week later, she developed mild blurring of vision with preserved reading ability, followed by transient numbness over the left frontal region. She was initially treated as migraine without improvement. Three weeks into the illness, she developed diplopia, predominantly on upward gaze, with vertical displacement of images. Two days prior to admission, she developed left-sided ptosis, worsening blurred vision, and epiphora. There was no history of fever, vomiting, photophobia, limb weakness, or facial deviation.

Neurological examination revealed preserved higher mental functions and normal speech. Cranial nerve examination demonstrated partial left ptosis, diplopia, gaze-evoked nystagmus, and limitation of left ocular movements consistent with involvement of cranial nerves III, IV, and VI. Sensory impairment was present over the ophthalmic division of the left trigeminal nerve. Visual acuity was clinically preserved and fundoscopic examination was normal. No optic disc edema or optic atrophy was identified. Facial motor function was intact. Hearing was preserved. Bulbar examination was normal without dysarthria or dysphagia. Motor strength, deep tendon reflexes, coordination testing, sensory examination of the limbs, and gait assessment were unremarkable. The clinical findings localized the lesion to the left cavernous sinus region.

Investigations 

Neuroimaging

**Figure 1:**
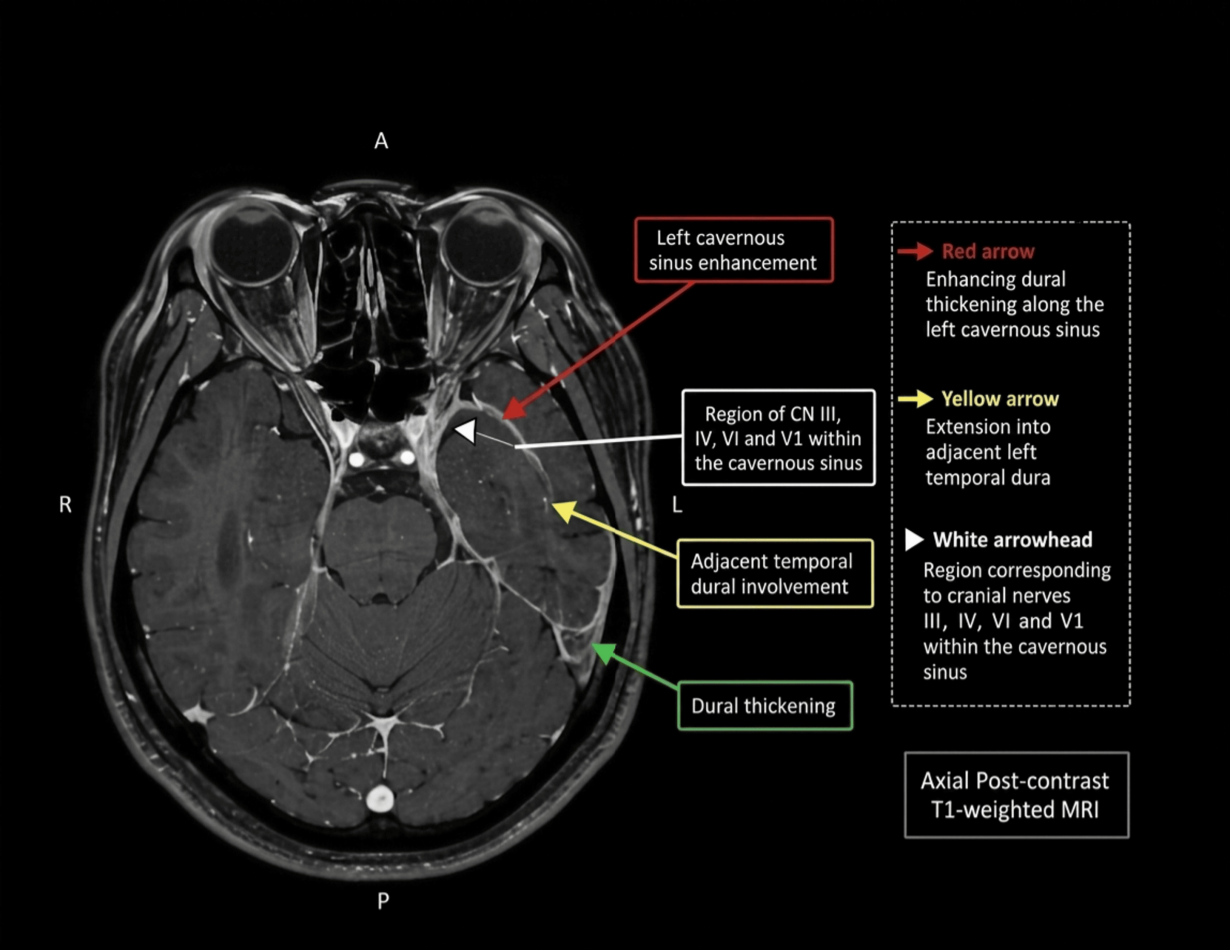
Contrast-enhanced MRI Brain, axial section, demonstrating hypertrophic pachymeningitis involving the left cavernous sinus. Axial post-contrast T1-weighted magnetic resonance imaging demonstrates abnormal enhancing dural thickening involving the left cavernous sinus (red arrow) with contiguous enhancement of the adjacent left temporal dura (yellow arrow). The lesion is located in the region traversed by cranial nerves (CN) III, IV, VI, and the ophthalmic division of cranial nerve V (white arrowhead), correlating with the patient’s ptosis, diplopia, ophthalmoplegia, and frontal sensory symptoms. The imaging findings are characteristic of hypertrophic pachymeningitis and, in conjunction with the exclusion of infectious, neoplastic, autoimmune, and vascular causes, supported the diagnosis of idiopathic hypertrophic pachymeningitis.

Magnetic resonance imaging of the brain with gadolinium contrast was performed to evaluate the cranial neuropathies. The imaging demonstrated abnormal enhancing dural thickening involving the left cavernous sinus and adjacent temporal region, corresponding anatomically to the patient's cranial neuropathies and supporting the diagnosis of IHP, as shown in Figure 1.

Cerebrospinal Fluid Analysis

An initial lumbar puncture attempt was complicated by a vasovagal episode associated with transient bradycardia and was aborted. A repeat lumbar puncture was subsequently completed under anesthesia. CSF analysis revealed no evidence of meningitis, malignant infiltration, or inflammatory pleocytosis. CSF biochemistry and cytology were unremarkable. Because hypertrophic pachymeningitis is fundamentally a diagnosis of exclusion, an extensive evaluation was undertaken.

Investigations for tuberculosis, cryptococcal infection, and fungal disease were negative. Fungal evaluation included beta-D-glucan testing. Serum protein electrophoresis, serum free light chain analysis, and kappa-lambda ratio were within normal limits. No evidence of systemic malignancy was identified clinically or radiologically. The absence of constitutional symptoms, systemic inflammatory manifestations, and laboratory evidence of infection or neoplasia supported an idiopathic inflammatory etiology. Although IgG4-related disease has increasingly been recognized as a cause of hypertrophic pachymeningitis, serum IgG4 testing was not available in this case. Because of the lesion location and the risks associated with biopsy, histopathological confirmation was not pursued. Following multidisciplinary review, a diagnosis of IHP was established.

Treatment 

The patient received intravenous methylprednisolone 1 g daily for five consecutive days, followed by a tapering dose of oral corticosteroids. During hospitalization,steroid-induced hyperglycemia was monitored and managed with insulin.

Outcome and follow-up

The patient demonstrated marked clinical improvement with substantial resolution of ophthalmoplegia, diplopia and ptosis. She was discharged on tapering steroids with antacids and calcium supplementation. Because relapse has been reported in patients with IHP, azathioprine was planned as a steroid-sparing agent pending thiopurine methyltransferase (TPMT) assessment. Long-term clinical follow-up was advised. Follow-up MRI imaging was not available at the time of manuscript preparation.

## Discussion

This case illustrates the diagnostic challenges posed by IHP, a rare fibro-inflammatory disorder of the dura mater. The radiological findings overlap with numerous infectious, inflammatory, vascular, and neoplastic conditions. Our patient initially presented with persistent unilateral frontotemporal headache and subsequently developed diplopia, ptosis, facial numbness, and ophthalmoplegia. The progressive involvement of cranial nerves III, IV, VI, and the ophthalmic division of cranial nerve V localized the lesion to the cavernous sinus region and prompted further neuroimaging.

The principal diagnostic challenge in this case was distinguishing IHP from other causes of painful ophthalmoplegia as IHP is a diagnosis of exclusion. Differential diagnoses included Tolosa-Hunt syndrome, cavernous sinus thrombosis, granulomatous diseases, vasculitic disorders, infectious pachymeningitis, neoplastic infiltration, and IgG4-related disease (Table 1) [3]. Contrast-enhanced MRI demonstrated focal enhancing dural thickening involving the left cavernous sinus and adjacent temporal dura, a finding characteristic of hypertrophic pachymeningitis. Extensive investigations failed to identify an infectious, neoplastic, autoimmune, or vascular etiology, supporting a diagnosis of IHP.

**Table 1:** Differential diagnosis with key clinical clues and distinguishing features TB PCR: Tuberculosis Polymerase Chain Reaction; c-ANCA PR3: Cytoplasmic Antineutrophil Cytoplasmic Antibody against Proteinase-3; ACE: Angiotensin-Converting Enzyme; Anti-CCP: Anti-Cyclic Citrullinatted Peptide Antibody; RA: Rheumatoid Arthritis Data synthesized from [3].

Differential Diagnosis	Reason for Consideration	Findings Against Diagnosis
Tuberculous pachymeningitis	Common cause of secondary pachymeningitis	CSF studies and clinical evaluation negative for tuberculosis
Fungal pachymeningitis	Can present with headache and cranial neuropathies	No microbiological or clinical evidence of fungal infection
IgG4-related disease	Recognized cause of hypertrophic pachymeningitis	No systemic manifestations; serum IgG4 unavailable
Granulomatosis with polyangiitis	May cause dural inflammation and cranial neuropathies	No clinical, laboratory, or radiological evidence of vasculitis
Neurosarcoidosis	Can mimic hypertrophic pachymeningitis	No systemic or imaging features suggestive of sarcoidosis
Malignancy (lymphoma/metastasis)	Dural enhancement may mimic neoplastic disease	Imaging and investigations did not support malignancy
Cavernous sinus thrombosis	Causes painful ophthalmoplegia and cranial neuropathies	MRI findings not consistent with thrombosis
Tolosa-Hunt syndrome	Important cause of painful ophthalmoplegia	Extent of dural thickening favored hypertrophic pachymeningitis
Idiopathic hypertrophic pachymeningitis	Diagnosis of exclusion	Supported by MRI findings, exclusion of secondary causes, and corticosteroid response

An important consideration was the distinction between orbital apex syndrome and cavernous sinus syndrome. Although the patient reported blurred vision, objective evidence of optic nerve dysfunction was not documented. Visual acuity remained clinically preserved, fundoscopic examination was normal, and no optic disc abnormalities were identified. Consequently, the clinical presentation was more accurately classified as cavernous sinus syndrome mimicking orbital apex pathology rather than true orbital apex syndrome.

This case emphasizes the importance of considering IHP in patients presenting with persistent unilateral headache and progressive painful cranial neuropathies. Early MRI evaluation and systematic exclusion of secondary causes are essential for timely diagnosis and initiation of treatment, which may prevent irreversible neurological deficits.MRI remains the cornerstone of diagnosis, typically demonstrating dural thickening with contrast enhancement [4].

The presence of gaze-evoked nystagmus represented an additional noteworthy finding. In the absence of radiological evidence of brainstem pathology, this finding was considered most likely secondary to ocular motor imbalance resulting from multiple cranial nerve dysfunction rather than direct central nervous system involvement.

Recent literature has demonstrated that a proportion of cases previously categorized as idiopathic may actually represent IgG4-related disease, highlighting the importance of thorough evaluation [5]. Histopathological confirmation is considered the diagnostic gold standard; however, biopsy is frequently impractical when lesions involve surgically inaccessible regions such as the skull base or cavernous sinus. In the present case, biopsy was not performed because of the lesion location and the patient’s rapid clinical improvement following corticosteroid therapy. Therefore, the diagnosis was based on characteristic MRI findings, exclusion of secondary causes, and a favorable therapeutic response.

Corticosteroids remain first-line therapy and frequently produce dramatic symptomatic improvement. Nevertheless, recurrence rates remain substantial in published series, highlighting the importance of long-term follow-up and consideration of steroid-sparing immunosuppressive agents. Azathioprine was selected as a potential maintenance therapy because of its established role in reducing relapse risk and minimizing cumulative corticosteroid exposure [6].

Compared with previously reported cases, this patient demonstrated relatively rapid progression from isolated headache to multiple cranial neuropathies localized to the cavernous sinus. The absence of systemic manifestations and the favorable response to corticosteroid therapy further support an inflammatory etiology and underscore the importance of maintaining a high index of suspicion for IHP in patients with painful ophthalmoplegia.

Limitations 

Several limitations should be acknowledged. Histopathological confirmation-biopsy was not obtained because of the anatomical location of the lesion and the favorable clinical response to corticosteroid therapy. Serum IgG4 testing was unavailable. Formal color vision testing, visual field assessment, and long-term radiological follow-up were not available at the time of manuscript preparation. Despite these limitations, the diagnosis was supported by characteristic clinical findings, MRI features, exclusion of secondary causes, and a robust therapeutic response to corticosteroids.

Learning points

Persistent unilateral headache accompanied by progressive cranial neuropathies should prompt evaluation for cavernous sinus pathology. IHP remains a diagnosis of exclusion and requires comprehensive investigation for infectious, autoimmune, neoplastic, and vascular causes. MRI with contrast is the imaging modality of choice and typically demonstrates enhancing dural thickening. Cavernous sinus involvement may closely mimic orbital apex pathology and other causes of painful ophthalmoplegia. Early corticosteroid therapy can result in substantial neurological recovery and may prevent irreversible deficits. Long-term follow-up is essential because disease recurrence is well recognized.

## Conclusions

IHP is a rare fibro-inflammatory disorder that may present with persistent unilateral headache and progressive cranial neuropathies due to cavernous sinus involvement. Because clinical and radiological findings overlap with infectious, autoimmune, neoplastic, and vascular disorders, the diagnosis remains one of exclusion and requires careful clinicoradiological correlation. Early contrast-enhanced MRI and systematic evaluation for secondary causes are essential for timely diagnosis. Although this patient demonstrated significant improvement following corticosteroid therapy, longer follow-up and radiological monitoring are necessary because recurrence is well recognized. This case highlights the importance of considering IHP in patients presenting with painful ophthalmoplegia and cavernous sinus syndrome.
